# Affectively effective: Work-related emotional intelligence as a predictor of organizational citizenship

**DOI:** 10.3389/fpsyg.2023.1092254

**Published:** 2023-02-08

**Authors:** Michael D. Robinson, Roberta L. Irvin, Sukumarakurup Krishnakumar

**Affiliations:** ^1^Psychology Department, North Dakota State University, Fargo, ND, United States; ^2^Henry E. Riggs School of Applied Life Sciences, Keck Graduate Institute of Applied Life Sciences, Claremont, CA, United States

**Keywords:** emotional intelligence, ability, work, contextual behavior, organizational citizenship

## Abstract

**Introduction:**

Efforts to link ability-related emotional intelligence to organizational behavior have resulted in modest findings.

**Methods:**

The present three studies examine whether a work-contextualized form of emotional intelligence (W-EI) may have greater predictive value, particularly in the organizational citizenship domain. Because W-EI should benefit social relationships within the workplace, positive associations between W-EI and organizational citizenship behavior were hypothesized.

**Results:**

This hypothesis was supported in three studies (total *N* = 462) involving samples of part-time student employees (Study 1), postdoctoral researchers (Study 2), and full-time employees (Study 3). All studies also provided evidence for incremental validity, such as with respect to the Big 5 personality traits, and Study 3 highlighted processes related to workplace engagement (in the form of higher levels of interpersonal job satisfaction and lower levels of burnout).

**Discussion:**

The results demonstrate the importance of W-EI in understanding employee variations in organizational citizenship.

## Introduction

As part of the “affective revolution” in organizational science, the idea that emotionally intelligent (EI) employees may be better employees has been of interest for several decades (Ashkanasy and Humphrey, 2011). Emotional intelligence can be assessed in trait-related terms or as an ability, but these streams of research need to be distinguished from each other (Mayer et al., 2008) because the two sorts of assessments do not correlate very highly with each other (Joseph and Newman, 2010) and/or assess fundamentally different constructs (Roberts et al., 2010). One rationale for such dissociations is that people have poor insight into their emotional abilities (Sheldon et al., 2014), and the present research is concerned with ability-related variations in emotional intelligence (ability EI) rather than self-reports of emotional ability (Mayer et al., 2008).

In the present research, we will begin by making the case that generic measures of ability EI display small or modest relationships with organizational outcomes (Ybarra et al., 2014). However, there are reasons for thinking that an ability EI measure that is contextualized for the workplace may fare better (Robinson et al., 2019). This point is investigated in the context of possible relationships between work-related emotional intelligence (W-EI) and organizational citizenship behaviors, which are non-mandatory behaviors that nonetheless play a large role in supporting organizational functioning (Werner, 2000). To obtain high W-EI scores, the test-taker needs to possess both emotion knowledge and social cognitive skills and this set of social–emotional skills is posited to contribute to better relationships in the workplace, which should be linked to higher levels of interpersonal citizenship behavior (Bowler and Brass, 2006). Partly as a consequence of such relationships, individuals with higher levels of W-EI should achieve higher levels of integration and engagement within the workplace, which would be linked to citizenship behaviors benefitting the organization as a whole. Possible relationships between W-EI and citizenship behaviors will be examined in three studies.

### The need for a contextualized measure

Efforts to link ability EI to organizational outcomes have resulted in modest findings. With respect to the performance of job-related tasks, Joseph and Newman (2010) reported a meta-analytic correlation of 0.16. However, ability EI did not predict task performance when controlling for personality plus cognitive ability and ability EI predicted task performance only in jobs that required high levels of emotional labor. O’Boyle et al. (2011) updated these meta-analytic conclusions and the results were similar. The ability EI/task performance correlation was 0.21, but this figure dropped to 0.07 when controlling for other individual difference factors. In additional meta-analyses, ability EI appears to be a largely inconsequential predictor of job satisfaction (Miao et al., 2017a), authentic leadership (Miao et al., 2018), and organizational citizenship behavior, particularly when controlling for other individual difference factors (Miao et al., 2017b). Findings such as these have led some commentators to conclude that ability EI may be a largely inconsequential predictor of workplace behaviors or outcomes (Zeidner et al., 2004; Ybarra et al., 2014).

Part of the problem is that the measures used in these studies—such as the MSCEIT (Mayer et al., 2003)—were not designed for the workplace. In fact, the MSCEIT includes tasks, such as ascribing emotions to abstract images or likening emotions to physical sensations, that would seem to possess little relevance to workplace functioning. This analysis is pertinent because the nature of a predictor will determine what it predicts (Hogan and Roberts, 1996) and measures contextualized for the workplace, relative to measures that are not, are likely to be better predictors of workplace outcomes (Lievens and De Soete, 2012). A general case for this point has been made in the attitude literature, which has shown that measures targeting particular behaviors predict those behaviors much better than more general (non-targeted) measures do (Ajzen and Timko, 1986; Kraus, 1995; Siegel et al., 2014). A more specific case for this point has been made in a literature that has shown that personality measures that have been contextualized for the workplace (such as by adding “at work” to items) predict workplace outcomes better than non-contextualized personality measures do (Bowling and Burns, 2010; Fisher et al., 2017). The predictive benefits of contextualization are likely to extend to many classes of predictors (Wernimont and Campbell, 1968), including those of an ability-related type (Lievens and De Soete, 2012).

On the basis of such considerations, Krishnakumar et al. (2016) created an ability-based EI measure for use in workplace settings (also see Schlegel and Mortillaro, 2019). The test—which is termed the NEAT (North Dakota Emotional Abilities Test)—uses a situational judgment test (SJT) format, both to assess social cognitive skills (Persich et al., 2020) and because SJT tests have been shown to be valid predictors of workplace outcomes (McDaniel et al., 2007). In the perception task, test-takers are asked to rate the extent to which an employee would experience a series of four emotions in a given scenario (e.g., “There have been widespread layoffs in Margie’s organization recently”). In the management task, test-takers are asked to rate the effectiveness of four different ways of responding to a situation that would induce emotions in its participants (e.g., “Stephan saw his co-worker struggling to a considerable extent”). The test is scored using a proportion consensus metric (Mayer et al., 2003) that utilizes the ratings of 82 workplace leaders, as described below. Krishnakumar et al. (2016) report extensive reliability and validity information for the NEAT and scores from the test are now referred to in terms of variations in work-related emotional intelligence (W-EI: Robinson et al., 2019).

### Investigating possible relationships with organizational citizenship

Thus far, the NEAT has primarily been used in studies focused on the prediction of workplace affect (e.g., Robinson et al., 2020), task performance (e.g., Krishnakumar et al., 2019), or deviance and/or counterproductive work behavior (e.g., Robinson et al., 2019). What remains to be known is whether employees with higher levels of W-EI are better organizational citizens. Findings of this type would represent an important discovery because organizational citizenship behaviors (OCBs) contribute to better ratings of job performance (Borman and Motowidlo, 1997) and benefit organizational functioning (Werner, 2000). Indeed, higher rates of OCB, which can be defined in terms of helpful and conscientious behaviors within the workplace (e.g., showing up on time, helping coworkers, and speaking well of one’s organization) have been linked to managerial ratings of performance, turnover intentions, productivity, efficiency, and organizational performance (Podsakoff et al., 2009). As an example of the benefits of OCB, Walz and Niehoff (2000) found that restaurants with higher OCB rates were more efficient, produced higher-quality service, and garnered higher ratings of customer satisfaction. In the present studies, we therefore focused squarely on this class of behaviors.

Organizational citizenship behaviors can be classified in various ways (Podsakoff et al., 2000), but the most stable distinction involves whether the behavior benefits individual targets (OCB-Is) or the organization as a whole (OCB-Os; Spitzmuller et al., 2008). Included in the first category would be helping coworkers who have been absent or taking time to listen to coworker concerns; included in the second category would be showing up on time and protecting organizational property (Williams and Anderson, 1991). There are reasons for thinking that higher levels of W-EI should be linked to a greater frequency of OCB-Is. Perhaps most straightforwardly, obtaining higher scores on the NEAT would seem to require skills related to perspective taking and empathetic concern—that is, the successful test taker must be capable of appreciating the plight of a protagonist and simulating the emotional reactions that he/she (i.e., the target character) would experience. Empathy and perspective taking, in turn, have been linked to prosocial behavior generally (Eisenberg and Miller, 1987; Batson, 1991; Van der Graaf et al., 2018) and to organizational citizenship behavior in particular (Borman et al., 2001; Joireman et al., 2006). This analysis accords with the results of several studies that have linked self-reported levels of emotional intelligence to perspective taking (Schutte et al., 2001), empathetic concern (Hajibabaee et al., 2018), and to relationship-enhancing behaviors that would tend to follow from perspective taking and empathetic concern (Schröder-Abe and Schütz, 2011).

Second, it is widely thought that facility with the emotion domain contributes to better interpersonal functioning (Halberstadt et al., 2001; Brackett et al., 2006; Farmer and Chapman, 2016). In fact, emotional intelligence is often considered a type of social intelligence (Mayer and Salovey, 1993; Schlegel et al., 2013) and individuals who can understand their emotions, and manage them in skillful manners, tend to have better interpersonal relationships (Schutte et al., 2001; Schröder-Abe and Schütz, 2011; Farmer and Chapman, 2016). In support of this point, a series of studies by Lopes and colleagues have linked the management branch of the MSCEIT to outcomes such as relationship quality, popularity, and lesser tendencies toward interpersonal conflict (Lopes et al., 2003, 2004, 2011). The NEAT, too, has been linked to perceptions of social support (Krishnakumar et al., 2019) and to satisfaction with interpersonal features of the workplace (Krishnakumar et al., 2016). Satisfaction with one’s workplace colleagues should, in turn, give rise to higher levels of organizational citizenship behavior (Spitzmuller et al., 2008; Organ, 2018). In concert with this proposed model, Study 3 will examine whether satisfaction with interpersonal features of the job mediates relationships between W-EI and OCB-I rates.

Beyond individual relationships, social and emotional skills can support higher levels of social integration, defined in terms of being an active, engaged member of a community (Berkman et al., 2000). Socially integrated individuals identify with the communities to which they belong and they enact a higher frequency of responsible behaviors as well as supportive reciprocal exchanges (Ware et al., 2007). In fact, Brañas-Garza et al. (2010) contend that there are evolutionary reasons for thinking that social integration supports prosocial behavior (Nowak, 2006) and Brañas-Garza et al. (2010) found that participants with higher levels of social integration allocated more money to strangers in a dictator game. Through the application of social and emotional skills within the workplace, the high W-EI employee should develop higher levels of social integration within their workplaces, which should give rise to higher levels of citizenship, particularly of an OCB-O type (Podsakoff et al., 2000; Chiu and Tsai, 2006). In support of the latter links, variables such as organizational identification (Lee et al., 2015) and organizational commitment (Meyer et al., 1989) are robust predictors of OCB-O rates (Podsakoff et al., 2000).

Within the workplace, social integration would support something termed work engagement, which can be defined in terms of investing oneself in one’s work role (Wefald et al., 2012). Investments of this type support job performance (Bailey et al., 2017), but they also support citizenship behaviors, particularly of an OCB-O type (Rich et al., 2010). Given that we have suggested that higher levels of W-EI should be linked to higher levels of social integration, they should support greater workplace engagement as well. Indirect evidence for this point would be evident to the extent that W-EI correlates positively with OCB-O rates, which suggest investment in one’s work role and the broader organizational culture (Macey and Schneider, 2008). More direct evidence would take the form of lower levels of workplace burnout, which is antithetical to engagement (Schaufeli and Bakker, 2004), and which may mediate relationships between W-EI levels and OCB-O rates (for a relevant precedent, see Swider and Zimmerman, 2010). Study 3 will examine mediation-related processes of this type.

Altogether, the present investigation consisted of three studies. In Study 1, we sought to examine relationships between W-EI and OCB among part-time (student) workers, who are an important component of the workforce (Conway and Briner, 2002). In Study 2, we reasoned that it would be good to obtain one sample of employees who held similar positions and obtained a large sample of postdoctoral researchers. Even in this context, we expected positive associations between W-EI and organizational citizenship behaviors. In Study 3, finally, we obtained a diverse sample of full-time employees and again predicted positive relationships between W-EI and OCB levels. In all studies, we focused on the distinction between individual-targeted OCBs and organization-targeted OCBs (Spitzmuller et al., 2008) and hypothesized relationships with both OCB types. All studies also focused on questions of incremental validity, for example with respect to personality traits such as agreeableness and conscientiousness (Sackett and Walmsley, 2014), and Study 3 additionally focused on mechanisms related to job satisfaction and burnout. In total, the investigation was designed to establish the relationships of interest while exploring additional questions about them.

## Study 1

### Method

#### Participants and procedures

We sought adequate (0.80) power to detect zero-order relationships in the 0.3 range, following precedent (Krishnakumar et al., 2016). G*Power software (Faul et al., 2009) recommended a sample size of 84 and we recruited 83 (48.19% female; 90.36% Caucasian; *M* age = 21.16) undergraduate students from a north Midwestern University in the United States who were working at least 20 h per week (of note, sample sizes exceeded 84 in Studies 2 and 3). Participants signed up for the study with SONA software and completed the study at a secure Qualtrics-programmed website, following which research credit was awarded. The average employee worked 26.52 h per week, had worked at their places of employment for 17.30 months, and types of employment included accounting, customer service, health care, manufacturing, office management, and sales. Data and a materials file for all studies can be found at OSF: https://osf.io/26tcu/?view_only=ec9f2275950c467ead7f4c16ee080451.

#### Work-related emotional intelligence

Work-related emotional intelligence was assessed with the NEAT, which applies the situational judgment method (Corstjens et al., 2017) to the key emotional intelligence tasks of perception, understanding, and management (Joseph and Newman, 2010), with a specific focus on workplace events and contexts (Krishnakumar et al., 2016). By embedding all scenarios and ways of responding within a workplace context, the hope is to capture a particular form of emotional reasoning that should have particular relevance to workplace functioning (Shaffer and Postlethwaite, 2012). In studies by Krishnakumar et al. (2016), the understanding branch of the NEAT tended to be less reliable and valid than the perception and management branches. In addition, the perception and understanding branches involve similar tasks—namely, rating the extent to which characters would experience particular emotions. Finally, the correlation among latent factors indicated that the perception and understanding branches were largely redundant (*r* = 0.81). For all of these reasons, and to support efficiently of measurement, we used a revised version of the NEAT that administers the perception and management tasks, but not the understanding task (Robinson et al., 2020).

The perception and management branches are thought to capture explicit and implicit (more behavioral) forms of emotion knowledge, complementing each other well (Robinson et al., 2019). The perception task asks individuals to rate the extent to which 10 protagonists (e.g., “Cassidy successfully finished a project that took months to accomplish”) would each experience four different emotions (e.g., joy and interest). The management task then asks individuals to rate the effectiveness of four different ways of responding (e.g., take over the co-worker’s more challenging tasks, hope the co-worker eventually “gets” it) to another 10 emotional situations (e.g., “Stephan saw his co-worker struggling to a considerable extent”).

For each task, responses range from poor ones to moderately good ones to very good ones, and both tasks were therefore paired with five-point rating scales (e.g., 1 = very ineffective; 5 = very effective). Ratings were then rescored using proportion consensus scoring metrics, which have performed well in many previous studies (e.g., Barchard et al., 2013). Participants, that is, received scores that reflected the percentage of an expert sample who made the same rating for a particular item (e.g., 0.2683 if 26.83% of the expert sample made the same rating that the participant did). Given the focus on workplace knowledge, expert norms were obtained from 82 workplace leaders (administrators, CEOs, etc.) who had an average of 18.53 years of workplace experience and an average of 27.15 supervisees. Scores were averaged across items for a particular scenario and then across scenarios to quantify work-related emotional intelligence (W-EI) in general terms (*M* = 0.3027; *SD* = 0.0625; α = 0.92). Of note, chance responding would produce a score of 0.2000 and the maximal possible score was 0.4531, which would be awarded if the participant always matched the highest percentage of expert raters for all of the ratings that they made. In actuality, scores ranged from 0.1083 to 0.3985. For comparison purposes, we also computed separable perception (*M* = 0.3286; *SD* = 0.0812; α = 0.92; range = 0.0614–0.4175) and management (*M* = 0.2768; *SD* = 0.0649; α = 0.90; range = 0.1456–0.4020) scores. The perception and management branches were correlated at *r* = 0.46, consistent with the presence of a general factor (Krishnakumar et al., 2016).

In previous studies, the NEAT has been shown to be a reliable measure (Krishnakumar et al., 2016; Robinson et al., 2019). It has also been shown to predict workplace performance (e.g., Krishnakumar et al., 2019) and deviant workplace behaviors (Robinson et al., 2019). In addition, the NEAT displays sensible correlations with personality, general mental ability, and other performance-based EI measures (Krishnakumar et al., 2016).

#### Organizational citizenship behaviors

Carpenter et al. (2014) have shown that self-reports of organizational citizenship behavior (OCB) are generally preferable to other-reports of the same behaviors, in part because employees have greater knowledge of their own behaviors than others (supervisors or coworkers) do (Allen et al., 2000). Furthermore, other-reports of OCB seem to lack incremental validity relative to self-reports of OCB (Carpenter et al., 2014). For such reasons, and because we sought to establish a novel relationship across multiple studies, we probed for tendencies toward OCB through the use of the self-report method. Of importance, relationships between W-EI and OCB cannot be ascribed to self-reports predicting self-reports because the W-EI measure is an ability-based one (Mayer et al., 2008).

Williams and Anderson (1991) provided support for the discriminability of OCBs with an individual target focus (e.g., helping a coworker) versus OCBs with an organizational target focus (e.g., conserving organizational resources) and this distinction has considerable merit to it (Spitzmuller et al., 2008). As a first way of assessing OCBs within the workplace, we therefore administered the Williams and Anderson (1991) scales. One seven-item scale focused on OCBs intended to help individuals (e.g., “I help others who have been absent”; *M* = 5.37; *SD* = 1.13; α = 0.90) and the other focused on OCBs intended to help the organization (e.g., “I conserve and protect organizational property”; *M* = 5.51; *SD* = 0.97; α = 0.69).

Another major OCB taxonomy was introduced by Organ (1988), who proposed the categories of altruism, conscientiousness, civic virtue, courtesy, and sportsmanship. In the present study, we focused on the first three categories relative to the second 2 because the first three categories implicate actions (e.g., arriving early to work) rather than inactions (e.g., not complaining). Employees were asked to indicate the extent to which they have engaged in altruistic behaviors (e.g., “I am willing to assist new colleagues to adjust to the work environment”; *M* = 4.10; *SD* = 0.73; α = 0.88), conscientious behaviors (e.g., “I often arrive early and start to work immediately”; *M* = 4.00; *SD* = 0.73; α = 0.86), and behaviors consistent with civic virtue (e.g., “I make constructive suggestions that can improve the operation of the company”; *M* = 3.91; *SD* = 0.74; α = 0.86), using scales developed by Podsakoff and MacKenzie (1994). Altruism fits into the OCB-I category and conscientious behavior and civic virtue fit into the OCB-O category (Podsakoff et al., 2000).

#### Additional variables

Participants reported on gender and job tenure. Additionally, they completed a factual autonomy scale (Spector and Fox, 2003) that probed for degrees of autonomy within the workplace (*M* = 3.94; *SD* = 0.78; α = 0.81). Such degrees of latitude could reasonably result in higher OCB rates, given that OCBs are defined in discretionary terms (Organ, 1988).

### Results

#### Initial results involving W-EI total scores

We hypothesized that employees with higher W-EI levels would engage in OCBs more frequently. This hypothesis was examined in five simple regressions, the results of which are displayed in Table 1. As shown in Table 1, W-EI predicted all forms of organizational citizenship, with Betas ranging from 0.28 to 0.56.

**Table 1:** Work-related emotional intelligence (W-EI) as a predictor of organizational citizenship behavior (OCB), simple regression results, Study 1.

OCB dimension and predictor	*t*	*p*	*β*
OCB-individual			
W-EI total score	4.39	<0.001	0.44
Perceptual EI	5.31	<0.001	0.51
Management EI	1.93	0.057	0.21
OCB-organizational			
W-EI total score	6.08	<0.001	0.56
Perceptual EI	5.14	<0.001	0.50
Management EI	4.64	<0.001	0.46
Altruism			
W-EI total score	5.01	<0.001	0.49
Perceptual EI	3.95	<0.001	0.40
Management EI	4.34	<0.001	0.43
Conscientiousness			
W-EI total score	4.21	<0.001	0.42
Perceptual EI	3.34	0.001	0.35
Management EI	3.71	<0.001	0.38
Civic Virtue			
W-EI total score	2.63	0.010	0.28
Perceptual EI	1.93	0.057	0.21
Management EI	2.60	0.011	0.28

Fifteen simple regressions were performed.

#### Branch-specific analyses

The skills assessed by the NEAT are probably best conceptualized in total-score (global EI) terms (Krishnakumar et al., 2016). Nonetheless, because we were interested in the possibility of unique relationships, we performed follow-up simple regressions that replaced the W-EI total score with one branch (perception or management) considered alone. These results, which are also displayed in Table 1, indicate that both perception scores (average β = 0.39) and management scores (average β = 0.35) predicted the occurrence of OCBs, probably because both branches require individuals to understand the emotional states of others, whether explicitly (perception) or implicitly (management).

#### Incremental validity

To bolster the case for discriminant or incremental validity, we performed five multiple regressions that included the predictors of W-EI (total score), gender (−1 = male; +1 = female), factual autonomy, and job tenure. As indicated in Table 2, the W-EI continuum remained a significant predictor of all forms of OCB with the other factors controlled.

**Table 2:** Work-related emotional intelligence (W-EI) as a predictor of organizational citizenship behavior (OCB), multiple regression results, Study 1.

OCB dimension and predictor	*t*	*p*	*β*
OCB-individual			
W-EI total score	4.36	<0.001	0.44
Gender	1.62	0.109	0.16
Factual autonomy	0.60	0.553	0.06
Job tenure	0.51	0.610	0.05
OCB-organizational			
W-EI total score	6.41	<0.001	0.55
Gender	2.80	0.006	0.24
Factual autonomy	3.49	<0.001	0.30
Job tenure	0.31	0.755	0.03
Altruism			
W-EI total score	4.99	<0.001	0.49
Gender	0.20	0.845	0.02
Factual autonomy	1.21	0.231	0.12
Job tenure	0.79	0.434	0.08
Conscientiousness			
W-EI total score	4.24	<0.001	0.42
Gender	0.26	0.794	0.03
Factual autonomy	2.34	0.022	0.23
Job tenure	0.70	0.486	0.07
Civic virtue			
W-EI total score	2.57	0.012	0.28
Gender	0.86	0.394	0.09
Factual autonomy	0.60	0.550	0.07
Job tenure	0.31	0.759	0.03

Five multiple regressions were performed. Gender was scored such that females received a higher score (−1 = male; +1 = female).

### Discussion

We hypothesized systematic relationships between W-EI and organizational citizenship behaviors. Study 1, which constituted an initial investigation of such relations, confirmed both that these associations were robust and that they were associated with large (Gignac and Szodorai, 2016) effect sizes. There was some indication that W-EI may be a stronger predictor of organizational forms of citizenship relative to individual-targeted forms of OCB, but both such relationships were evident. W-EI, therefore, seems to be an important predictor of organizational citizenship, pending replication.

## Study 2

Study 2 sought to replicate relations between W-EI and organizational citizenship behaviors in the context of a single occupation, which would control for several factors that could vary across jobs. Study 2 did so by recruiting a sample of postdoctoral researchers, who occupy a sort of limbo between graduate studies and longer-term positions that are aspired to (Akerlind, 2005). In addition, we collected self-reports of personality, which would allow us to demonstrate incremental validity with respect to personality.

### Method

#### Participants and procedures

The Study 2 sample consisted of post-doctoral researchers who were recruited in two manners. Initially, a Research Assistant visited the websites of major departments in the life sciences (biology, molecular biology, biochemistry, plant sciences, biological sciences, wildlife biology, and ecology) throughout the United States, which led to a compiled list of 1,076 postdoctoral researchers. These individuals were emailed and asked whether they would be willing to complete a study concerning their postdoctoral experiences in return for a chance to win a $20 gift card. Forty-eight individuals used the provided link to complete the Qualtrics-programmed survey on a secure website. Additionally, we obtained an email list of approximately 3,400 postdoctoral researchers from the National Post-Doctoral Association. These individuals were also invited to complete the postdoctoral experiences study over the Internet, in return for a chance to win a $20 gift card. Usable surveys (following from this second recruitment effort) were completed by 184 participants, for a total of 232 participants.

The vast majority (98.71%) of postdoctoral researchers worked in university settings in biological science disciplines. The sample was 65.09% female with a mean age of 32.86 (65.09% Caucasian, 24.14% Asian, 4.31% Hispanic, 3.45% African American, and 3.02% Pacific Islander). Participants had been in their current postdoctoral positions for an average of 24.23 months and the average job salary was $46,107. Participants completed the NEAT and demographic information prior to reporting on their behaviors within the postdoctoral setting.

#### Work-related emotional intelligence

Work-related emotional intelligence was again assessed with the NEAT, which was designed to capture skills and abilities pertinent to the workplace context (Krishnakumar et al., 2016). The perception task required individuals to discern the emotions that would be present in different situations (*M* = 0.3401; *SD* = 0.0648; α = 0.89; range = 0.1516–0.4296) and the management task required individuals to indicate which sorts of responses would be most effective in another set of emotion-laden situations (*M* = 0.3037; *SD* = 0.0640; α = 0.76; range = 0.1558–0.3946). Of most interest was the W-EI total score (*M* = 0.3219; *SD* = 0.0453; α = 0.85; range = 0.1860–0.4026), but we also retained perception and management scores for follow-up analyses.

#### Organizational citizenship behaviors

We assessed tendencies toward organizational citizenship in two manners. The distinction between OCBs that target individuals versus OCBs that target the organization is a good one (Spitzmuller et al., 2008) and we therefore administered the Williams and Anderson (1991) scales also administered in Study 1 (OCB-I: *M* = 5.33; *SD* = 0.90; α = 0.83; OCB-O: *M* = 5.50; *SD* = 0.75; α = 0.66).

Participants also characterized their contextual behavior, which are behaviors closely aligned with OCBs (Organ, 2018), in terms of the contextual behavior scales of Van Scotter and Motowidlo (1996). One subscale focused on interpersonal facilitation (e.g., “support or encourage a co-worker with personal problems”; *M* = 5.83; *SD* = 0.76; α = 0.81) and the other focused on job dedication (e.g., “put in extra hours to get work done on time”; *M* = 5.77; *SD* = 0.71; α = 0.80). Interpersonal facilitation is OCB-I-like in nature and job dedication is OCB-O-like in nature (Podsakoff et al., 2000).

#### Additional variables

Participants reported on gender and job tenure. In addition, we sought to include measures of cognitive ability and personality. Cognitive ability was assessed through the proxy of college GPA (*M* = 3.58; *SD* = 0.34) and the Big 5 personality traits were assessed with the Ten-Item Personality Inventory (TIPI: Gosling et al., 2003). The latter scales of extraversion (*M* = 3.78; *SD* = 1.60; α = 0.70), agreeableness (*M* = 5.22; *SD* = 1.19; α = 0.31), conscientiousness (*M* = 5.67; *SD* = 1.13; α = 0.52), neuroticism (*M* = 3.25; *SD* = 1.34; α = 0.63), and openness to experience (*M* = 5.25; *SD* = 1.06; α = 0.35) were brief, but TIPI scales have been shown to correlate highly with longer, typically more reliable, measures of the Big 5 (Ehrhart et al., 2009). Of note, W-EI levels shared positive relationships with agreeableness, *r* = 0.38, *p* < 0.001, and conscientiousness, *r* = 0.39, *p* < 0.001, which is a personality profile that has been linked to better organizational citizenship (Sackett and Walmsley, 2014).

### Results

#### Initial results involving W-EI total scores

As in Study 1, we hypothesized that employees with higher W-EI levels would engage in OCBs more frequently. This hypothesis was initially examined in 4 simple regressions, the results of which are displayed in Table 3. As shown there, W-EI was a positive predictor of all four forms of organizational citizenship, with Betas ranging from 0.34 to 0.43.

**Table 3:** Work-related emotional intelligence (W-EI) as a predictor of organizational citizenship behavior (OCB), simple regression results, Study 2.

OCB dimension and predictor	*t*	*p*	*β*
OCB-individual			
W-EI total score	5.42	<0.001	0.34
Perceptual EI	3.89	<0.001	0.25
Management EI	4.97	<0.001	0.31
OCB-organizational			
W-EI total score	7.13	<0.001	0.43
Perceptual EI	4.06	<0.001	0.26
Management EI	8.09	<0.001	0.47
Job dedication			
W-EI total score	5.91	<0.001	0.36
Perceptual EI	4.30	<0.001	0.27
Management EI	5.27	<0.001	0.33
Interpersonal facilitation			
W-EI total score	7.26	<0.001	0.43
Perceptual EI	6.21	<0.001	0.38
Management EI	5.27	<0.001	0.33

Twelve simple regressions were performed.

#### Branch-specific analyses

Skills related to perception and management correlated moderately in Study 2, *r* = 0.31, *p* < 0.001. It therefore made sense to perform follow-up regressions in which the predictive ability of each branch was considered separately. As shown in Table 3, all of the regressions involving perception were significant and all of the regressions involving management were also significant. Thus, both branches appear to be linked to organizational citizenship behaviors.

#### Incremental validity

To demonstrate incremental validity, we conducted four multiple regressions, one for each of the four outcomes. The predictors were W-EI total scores, gender (−1 = male; +1 = female), tenure, GPA, and all five dimensions of personality. As shown in Table 4, W-EI continued to predict all 4 OCB outcomes (examined in Study 2) when controlling for other factors. Relationships between W-EI and OCB were more consistent than relationships between personality and OCB.

**Table 4:** Work-related emotional intelligence (W-EI) as a predictor of organizational citizenship behavior (OCB), multiple regression results, Study 2.

OCB dimension and predictor	*t*	*p*	*β*
OCB-individual			
W-EI total score	3.31	0.001	0.27
Gender	1.33	0.186	0.10
Tenure	−0.12	0.901	−0.01
GPA	−1.38	0.169	−0.09
Extraversion	2.99	0.003	0.19
Agreeableness	2.54	0.012	0.18
Conscientiousness	−0.48	0.632	−0.04
Neuroticism	0.19	0.846	0.01
Openness to experience	0.03	0.974	0.00
OCB-organizational			
W-EI total score	3.89	<0.001	0.28
Gender	−1.60	0.111	−0.11
Tenure	0.07	0.947	0.00
GPA	−0.70	0.488	−0.04
Extraversion	−0.02	0.981	−0.00
Agreeableness	3.31	0.001	0.21
Conscientiousness	4.66	<0.001	0.32
Neuroticism	−0.41	0.684	−0.03
Openness to experience	1.55	0.123	0.10
Job dedication			
W-EI total score	2.90	0.004	0.22
Gender	0.85	0.396	0.06
Tenure	−0.35	0.727	−0.02
GPA	−1.68	0.095	−0.11
Extraversion	1.87	0.063	0.11
Agreeableness	−0.28	0.779	−0.02
Conscientiousness	4.48	<0.001	0.32
Neuroticism	−2.48	0.014	−0.16
Openness to experience	0.75	0.457	0.05
Interpersonal facilitation			
W-EI total score	4.65	<0.001	0.35
Gender	1.18	0.239	0.08
Tenure	0.66	0.511	0.04
GPA	−1.38	0.168	−0.09
Extraversion	3.97	<0.001	0.24
Agreeableness	2.52	0.012	0.17
Conscientiousness	0.95	0.341	0.07
Neuroticism	0.14	0.893	0.01
Openness to experience	−0.20	0.845	−0.01

Four multiple regressions were performed. Gender was scored such that females received a higher score (−1 = male; +1 = female).

### Discussion

The W-EI/OCB relationship remained stable among a sample of full-time employees. Betas for initial analyses were in the 0.34–0.43 range and both perception and management predicted these behaviors. Of importance, these relationships remained significant when controlling for personality and cognitive ability, which are individual differences that figure prominently in the organizational literature (Sackett and Walmsley, 2014). The skills and abilities tapped by W-EI can therefore be considered important ones, both with respect to contextual behaviors in general and OCBs in particular.

## Study 3

In Study 3, we sought to replicate W-EI/OCB relationships among a more heterogeneous sample of full-time employees, relative to Study 2. Study 1 had suggested that W-EI may be more strongly linked to organization-focused OCBs than dyad-focused OCBs and Study 2 also displayed small trends along these lines (see Tables 1, 3). Accordingly, we revisited this OCB-type distinction in the third study. Additionally, we suggested that W-EI should support higher levels of social engagement or integration, which should be evident in terms of higher levels of job satisfaction as well as lower levels of burnout (Rich et al., 2010). Further, such factors and processes matter for OCB rates (Podsakoff et al., 2000) and might therefore explain some of the variance linking W-EI to organizational citizenship. With respect to these analyses, though, we should caution that cross-sectional forms of mediation might or might not provide insights into longitudinal relationships among the variables (MacKinnon and Fairchild, 2009). Because this is true, mediational analyses should be considered supplemental rather than primary.

### Method

#### Participants and procedures

In Study 3, we contracted with Qualtrics, who are a top survey research company, to obtain a high-quality sample of 150 full-time employees. We specified that the employees needed to be working in the United States and that they needed to be 25 years old or older, which would target individuals working in their intended careers. We also specified an even mix of male and female participants. Qualtrics used their panel recruitment resources to target qualified employees from diverse occupations and respondents received points or credit as the result of their participation.

Qualtrics ensured that participants were eligible (e.g., residence in the US) and they also deleted responses from anyone failing any of four attention checks. We deleted another three individuals who completed the study too quickly (< 10 min) and this resulted in a final sample size of 147. A slight majority of the sample were married (54.62%) and the average age was 42.78. The sample was somewhat diverse with respect to gender (48.85% female), ethnicity (71% Caucasian, 12.98% African American, 9.16% Hispanic, and 4.58% Asian American), and geographic location, as participants resided in 42 different states. Jobs were also diverse and they included accountant, caregiver, educator, janitor, IT supervisor, registered nurse, wholesale parts manager, etc. The average job tenure was 11.34 years and the average annual salary was $61,078. The study was completed over the Internet, using Qualtrics software.

#### Work-related emotional intelligence

Work-related emotional intelligence was assessed with the NEAT (Krishnakumar et al., 2016), consistent with prior studies. Our primary interest was in W-EI defined in total terms (*M* = 0.3175; *SD* = 0.0500; α = 0.89; range = 0.1249–0.4015), but we also computed separable scores for perception (*M* = 0.3501; *SD* = 0.0569; α = 0.84; range = 0.1300–0.4225), and management (*M* = 0.2849; *SD* = 0.0584; α = 0.86; range = 0.1199–0.3826), which were correlated at *r* = 0.53.

#### Organizational citizenship behaviors

We began assessing organizational citizenship behaviors in a manner parallel to prior studies. Specifically, employees were asked to characterize their rates of individual-oriented OCBs (*M* = 5.50; *SD* = 1.09; α = 0.87) and organization-oriented OCBs (*M* = 5.54; *SD* = 1.04; α = 0.74), using the Williams and Anderson (1991) scales. We also administered the scales of McNeely and Meglino (1994) as a way of gaining further insights into W-EI/OCB relationships. There were seven items targeting prosocial organizational behavior (e.g., “speak favorably about the organization to outsiders”; *M* = 4.05; *SD* = 0.76; α = 0.88), seven targeting role-prescribed prosocial behavior (e.g., “arrive to work on time”; *M* = 4.33; *SD* = 0.62; α = 0.86), and six describing prosocial individual behaviors (e.g., “bring food to share with co-workers”; *M* = 3.17; *SD* = 1.07; α = 0.90). The last set of behaviors were somewhat specific and perhaps overly sentimental, but the subscale was included for the sake of complete reporting. Broadly speaking, prosocial organizational behavior and role-prescribed prosocial behavior fit into the OCB-O category and prosocial individual behavior fits into the OCB-I category (Podsakoff et al., 2000).

#### Potential mediators

We were interested in further understanding the reasons why employees with higher W-EI levels engage in OCBs more frequently. Such reasons, it seemed to us, are likely to involve affective and motivational processes that tie the individual to the workplace. That is, W-EI may facilitate more engagement and satisfaction in the workplace, which may, in turn, give rise to higher rates of OCB. To examine questions of this type, we asked employees to report on their satisfaction with interpersonal features of the job and we also asked them to report on experiences of burnout, which have been linked to lower levels of OCB in several studies (e.g., Chiu and Tsai, 2006).

Satisfaction with interpersonal features of the job was assessed by combining the satisfaction with coworkers and satisfaction with supervisor subscales of job satisfaction scale of Spector (1997). Employees responded to each of the eight relevant items (e.g., “I enjoy my coworkers”) and we computed a total score by averaging across items (*M* = 4.68; *SD* = 1.08; α = 0.88). Experiences of burnout were assessed with the work-related burnout scale of the Copenhagen Burnout Inventory (Kristensen et al., 2005), which has performed well in a number of studies (e.g., Fiorilli et al., 2015). Individuals responded to the seven relevant questions (e.g., “do you feel burnt out because of your work?”) and we averaged across ratings (*M* = 2.56; *SD* = 0.94; α = 0.88).

#### Personality assessment

The personality traits of the Big 5 were assessed using scales developed by Donnellan et al. (2006). Participants reported on the extent to which they could be characterized in terms of statements targeting extraversion (*M* = 3.25; *SD* = 0.89; α = 0.65), agreeableness (*M* = 3.79; *SD* = 0.79; α = 0.68), conscientiousness (*M* = 3.98; *SD* = 0.76; α = 0.62), neuroticism (*M* = 2.63; *SD* = 0.90; α = 0.63), and openness (*M* = 3.69; *SD* = 0.90; α = 0.71). Participants achieving higher W-EI scores were more extraverted, *r* = 0.17, *p* = 0.035, agreeable, *r* = 0.39, *p* < 0.001, conscientious, *r* = 0.39, *p* < 0.001, and open to experience, *r* = 0.32, *p* < 0.001. They were also lower in neuroticism, *r* = −0.28, *p* = 0.001.

### Results

#### Initial results involving W-EI total scores

Simple regressions were performed to determine whether there were positive relationships between W-EI levels and organizational citizenship behaviors (see Table 5). Considering the Williams and Anderson (1991) outcomes first, W-EI was a significant predictor of individual-targeted OCBs (β = 0.17) and a larger-magnitude predictor of organization-targeted OCBs (β = 0.59). Considering the McNeely and Meglino (1994) outcomes second, W-EI was a significant predictor of prosocial organizational behavior (β = 0.26) and role-prescribed prosocial behavior (β = 0.40), but it was a non-significant predictor of individual-level behaviors (e.g., bringing cake to work) that we characterized as overly sentimental (β = −0.13).

**Table 5:** Work-related emotional intelligence (W-EI) as a predictor of organizational citizenship behavior (OCB), Simple regression results, study 3.

OCB dimension and predictor	*t*	*p*	*β*
OCB-individual			
W-EI total score	2.09	0.039	0.17
Perceptual EI	2.35	0.020	0.19
Management EI	1.31	0.193	0.11
OCB-organizational			
W-EI total score	8.72	<0.001	0.59
Perceptual EI	6.23	<0.001	0.46
Management EI	8.23	<0.001	0.56
Prosocial OB			
W-EI total score	3.22	0.002	0.26
Perceptual EI	2.99	0.003	0.24
Management EI	2.61	0.010	0.21
Role-prescribed PB			
W-EI total score	5.21	<0.001	0.40
Perceptual EI	4.79	<0.001	0.37
Management EI	4.14	<0.001	0.32
Prosocial IB
W-EI total score	−1.63	0.106	−0.13
Perceptual EI	−0.51	0.614	−0.04
Management EI	−2.33	0.021	−0.19

Fifteen simple regressions were performed. Prosocial OB, prosocial organizational behavior; Role-Prescribed PB, role-prescribed prosocial behavior; Prosocial IB, prosocial individual behavior.

#### Branch-specific analyses

As shown in Table 5, perceptual abilities (as assessed by the NEAT) were predictive of four of the five outcomes, with the exception being the same one noted above (i.e., prosocial individual behavior, as assessed by the McNeely and Meglino, 1994, scale). Management abilities, considered alone, predicted the OCB-O, prosocial organizational behavior, and role-prescribed prosocial behavior outcomes. Although perceptual abilities tended to predict individual-targeted OCBs more positively, and management abilities tended to predict organization-targeted OCBs more positively, these were not very strong trends. Rather, the relevant ability sets tended to converge in their predictions.

#### Incremental validity

To preserve journal space, we will omit any further results involving prosocial individual behavior (McNeely and Meglino, 1994). For the remaining outcomes, we performed follow-up multiple regressions in which W-EI and all five personality dimensions were simultaneously controlled (Table 6). W-EI remained a significant predictor of the OCB-O and role-prescribed prosocial outcomes with all dimensions of the Big 5 controlled, but not the OCB-I or prosocial organizational behavior outcomes. Nonetheless, the average Beta coefficient for W-EI (average β = 0.19) was higher than that for extraversion (average β = 0.15), agreeableness (average β = 0.17), conscientiousness (average β = 0.15), neuroticism (average β = −0.03), and openness (average β = 0.02).

**Table 6:** Work-related emotional intelligence (W-EI) as a predictor of organizational citizenship behavior (OCB), multiple regression results, Study 3.

OCB dimension and predictor	*t*	*p*	*β*
OCB-individual			
W-EI total score	−0.03	0.977	−0.00
Extraversion	3.58	<0.001	0.29
Agreeableness	3.62	<0.001	0.35
Conscientiousness	−0.19	0.852	−0.02
Neuroticism	1.12	0.266	0.08
Openness to experience	0.56	0.578	0.05
OCB-organizational			
W-EI total score	5.93	<0.001	0.43
Extraversion	0.76	0.447	0.06
Agreeableness	1.18	0.240	0.10
Conscientiousness	2.53	0.013	0.21
Neuroticism	−0.44	0.664	−0.03
Openness to experience	0.63	0.532	0.05
Prosocial OB			
W-EI total score	1.24	0.219	0.11
Extraversion	2.07	0.040	0.18
Agreeableness	0.88	0.381	0.09
Conscientiousness	1.23	0.220	0.12
Neuroticism	−1.42	0.157	−0.12
Openness to experience	0.05	0.964	0.00
Role-prescribed PB			
W-EI total score	2.62	0.010	0.21
Extraversion	0.79	0.429	0.07
Agreeableness	1.38	0.170	0.14
Conscientiousness	2.97	0.004	0.27
Neuroticism	−0.83	0.409	−0.06
Openness to experience	−0.19	0.847	−0.02

Five multiple regressions were performed. Prosocial OB, prosocial organizational behavior; Role-Prescribed PB, role-prescribed prosocial behavior; and Prosocial IB, prosocial individual behavior.

#### Mediational pathways

W-EI was a positive predictor of satisfaction with interpersonal features of the job, *r* = 0.35, *p* < 0.001, and it was a negative predictor of burnout, *r* = −0.23, *p* = 0.006. Job satisfaction positively predicted all four OCBs (OCB-I: *r* = 0.24, *p* = 0.004; OCB-O: *r* = 0.42, *p* < 0.001; prosocial OB: *r* = 0.54, *p* < 0.001; role-prescribed PB: *r* = 0.35, *p* < 0.001) and burnout negatively predicted all four OCBs (OCB-I: *r* = −0.26, *p* = 0.002; OCB-O: *r* = −0.35, *p* < 0.001; prosocial OB: *r* = −0.29, *p* < 0.001; role-prescribed PB: *r* = −0.22, *p* = 0.006). Hence, it is plausible that both satisfaction with interpersonal features of the job and burnout could account for some of the variance linking W-EI to organizational citizenship.

To examine such questions, we performed eight mediation-related analyses using the PROCESS macro for SAS (Hayes, 2013), which employs bootstrapping methods to examine the significance of indirect pathways (MacKinnon and Fairchild, 2009). Four analyses examined possible mediation by satisfaction with interpersonal features of the job and four analyses examined possible mediation by burnout. Within each set, the four successive models focused on the OCB-I, OCB-O, prosocial organizational behavior, and role-prescribed prosocial behavior outcomes, respectively. As shown in Table 7, zero-order relationships between W-EI and each of the outcomes (*c* coefficient) were reduced in magnitude (*c’* coefficient) when accounting for the relevant mediational pathway (*ab*). Furthermore, all mediational pathways were significant, as indicated by the Bias Corrected Confidence Interval (BCCI) estimates for indirect pathways, which always excluded 0. Thus, satisfaction with interpersonal features of the job and burnout seem to play some role in linking W-EI to OCBs.

**Table 7:** Mediation results involving job satisfaction (interpersonal features) and burnout, Study 3.

Model	*a*	*b*	*c*	c’	BCCI for *ab* pathway
W-EI → JS-I → OCB-I	0.35*	0.20*	0.17*	0.10	0.01–0.16
W-EI → JS-I → OCB-O	0.35*	0.25*	0.59*	0.50*	0.03–0.16
W-EI → JS-I → Prosocial	0.35*	0.39*	0.26*	0.12	0.09–0.21
W-EI → JS-I → Role	0.35*	0.24*	0.40*	0.31*	0.04–0.16
W-EI → Burnout → OCB-I	−0.23*	−0.23*	0.17*	0.12	0.02–0.11
W-EI → Burnout → OCB-O	−0.23*	−0.23*	0.59*	0.54*	0.02–0.10
W-EI → Burnout → Prosocial	−0.23*	−0.25*	0.26*	0.20*	0.02–0.11
W-EI → Burnout → Role	−0.23*	−0.14	0.40*	0.37*	0.01–0.08

Eight investigations of mediation were conducted. W-EI, Work-Related Emotional Intelligence; JS-I, Job Satisfaction-Interpersonal Features; Prosocial, prosocial organizational behavior; and Role, role-prescribed prosocial behavior; *p < 0.05.

### Discussion

Study 1 had suggested that W-EI could be a stronger predictor of OCB-Os than OCB-Is and Study 3 seems to confirm this pattern. Indeed, the one scale that consisted of prosocial behaviors that have little organizational merit (e.g., bringing cake to work or sending birthday greeting cards to fellow employees) was the one OCB scale that did not correlate with the W-EI dimension. In further work on the W-EI/OCB interface, therefore, it may be useful to distinguish merely “nice” behaviors from those that are more central to organizational functioning. In support of such ideas, W-EI was positively linked to job satisfaction and negatively linked to workplace burnout (both of which are key organizational variables: Organ, 2018) and these relationships played some role in explaining W-EI/OCB relationships. The reduction in variance for OCB-O behaviors was slight, however, suggesting the need to consider other potential manifestations of W-EI such as social network centrality (Brañas-Garza et al., 2010).

## General discussion

Just as personality measures that have been contextualized for the workplace outperform those that do not (in predicting workplace outcomes: Shaffer and Postlethwaite, 2012), ability EI measures that that been contextualized for the workplace may do a better job of predicting behavior and performance at work. On the basis of such reasoning as well as reasoning linking workplace EI to higher levels of social integration, we hypothesized that a work-contextualized ability EI measure (the NEAT: Krishnakumar et al., 2016) would be a consistent positive predictor of organizational citizenship behaviors (OCBs). Such relations were evident across several sample types and they were also evident across four OCB taxonomies—those of Williams and Anderson (1991), Organ (1988), Van Scotter and Motowidlo (1996), and McNeely and Meglino (1994). The one exception was the prosocial individual behavior scale of McNeely and Meglino (1994), which focuses on sentimental behaviors that possess little organizational relevance. On the basis of the results, we conclude that W-EI is a robust predictor of performing contextual behaviors that benefit the organization.

Consistent with our theorizing with respect to social integration (Berkman et al., 2000), W-EI proved to be a stronger predictor of OCB-O behaviors (Betas = 0.56, 0.43, and 0.59 across Studies 1–3, respectively) than OCB-I behaviors (Betas = 0.44, 0.34, and 0.17). The skills and abilities assessed by W-EI may therefore tie the individual to the organization in ways that are respectful and conscientious to a somewhat greater extent than they result in prosocial behaviors of an individualized type. One way of understanding these findings is to suggest that the skills of individuals with higher W-EI levels lead them to become more integrated with the workplace, in organizational terms, resulting in commitment to behaviors that benefit the organization as a whole. Relatedly, we have suggested that high W-EI employees are more capable of sustaining workplace engagement, which would be linked to behaviors and practices of an OCB-O type (Rich et al., 2010). These ideas require further analysis, but a point worth making is that being a conscientious employee (or a good organizational citizen) is something that one can always do (Organ, 2018). By contrast, individualized citizenship behaviors may largely depend on coworkers having problems, whether personal or work-related (McNeely and Meglino, 1994). Such problems may only occasionally occur, resulting in fewer opportunities for resolving them.

Another angle that was pursued was whether OCBs might follow from the perception branch of W-EI, the management branch, or both. Perceptual skills, in particular, might support empathy and situational awareness (Schlegel and Scherer, 2016), but management skills should be more proximate to behavior (Joseph and Newman, 2010). In fact, perception (average β = 0.31) and management (average β = 0.30) proved to be equally predictive of OCBs. Furthermore, the strongest predictions were obtained when averaging across the perception and management branches (average β = 0.36). Thus, it may be what is common to perception and management (global EI) that matters the most.

In summarizing the findings, we emphasize several strengths. We were able to replicate W-EI/OCB relationships across several sample types—part-time employees (Study 1), full-time employees within a particular occupation (Study 2), and full-time employees from diverse occupations (Study 3). Also, W-EI/OCB relationships tended to remain significant when controlling for all of the personality traits captured by the Big 5 taxonomy and this was certainly true with respect to the most organizational types of citizenship behavior. Finally, we provided some evidence—in Study 3—for the idea that variables related to social affiliation and engagement provide some insights into why employees with higher levels of W-EI tended to be better organizational citizens, though the cross-sectional forms of mediation that were performed should be considered provisional until they are replicated in longitudinal analyses (MacKinnon and Fairchild, 2009; O’Laughlin et al., 2018). In the remainder of the General Discussion, we further explore implications and future directions.

### Further implications

The results provide further evidence in favor of the NEAT perspective on ability EI within the workplace. The NEAT embeds traditional EI tasks (such as perception and management) into a situational judgment test format and this format permits a consistent focus on workplace events and experiences (Krishnakumar et al., 2016). Previous results had indicated that W-EI, as assessed by the NEAT, is inversely linked to the probability of counterproductive work behavior (Krishnakumar et al., 2017) and deviance (Robinson et al., 2019). The current results additionally establish that employees with high W-EI levels are more inclined toward conscientious and helpful behaviors within the workplace. In total, then, W-EI seems to support better contextual performance both through the inhibition of undesirable workplace behaviors and through increased rates of desirable workplace behaviors. This is a profile that attests to the potential importance of the relevant individual differences, perhaps in contrast to previous investigations focused on ability EI, which have tended to use ability EI measures not designed for the workplace (see Schlegel and Mortillaro, 2019, for further considerations along these lines).

In this connection, it is common to ask questions about whether ability EI might support higher levels of well-being, either within or outside the workplace. Such relationships are often slight (MacCann et al., 2020) and the more profitable questions may relate to the manner in which individual differences in EI manifest themselves behaviorally, particularly in social contexts. Perceiving emotions accurately, for example, may sometimes result in higher levels of distress, particularly when concurrent circumstances are stressful (Engelberg and Sjöberg, 2004). Still, the abilities involved should render one more sensitive to the needs of others, which should be linked to cooperative social behavior (Halberstadt et al., 2001), and they should facilitate the skill with which the relevant behaviors can be performed (Farmer and Chapman, 2016). In this context, we emphasize the socio-emotional interface as a basis for further theorizing, much as Halberstadt et al. (2001) do.

There is certainly some relationship between W-EI and the personality trait of agreeableness, which involves cooperative social behavior and effectiveness in social contexts (Jensen-Campbell et al., 2010). However, there is also a relationship between W-EI and conscientiousness (e.g., see Study 3), which has been linked to more effective workplace behaviors more generally (Sackett and Walmsley, 2014). This combination of personality attributes can be thought of in terms of agentic communion (Mansfield and McAdams, 1996), maturity (DeYoung, 2010), or social competence (Gurtman, 1999). By this analysis, one would expect W-EI to support effective mentoring relationships, social communication, and at least certain forms of leadership, which are directions worthy of future research (Côté, 2014). Regardless, we emphasize the fact that the skills and abilities involved are not isomorphic with personality traits and many W-EI/OCB relationships remained significant when controlling for them.

The results, also, provide insights into the factors that give rise to employee variations in OCB rates. It is common to emphasize OCB antecedents that are conceptualized in situational (or job-specific) terms, such as organizational support or procedural justice (Spitzmuller et al., 2008). Independent of such factors, it appears that individual differences, at least of a certain type, matter quite a bit in predicting OCB rates. These individual differences relate to social–emotional skills (Mayer et al., 2016) or emotion knowledge (Izard et al., 2001) that matter for relationships and/or allow the individual to achieve higher levels of integration with the groups and organizations to which they belong (Ware et al., 2007). Bowler and Brass (2006) have shown that relationship ties powerfully predict OCB-I rates and a similar analysis, perhaps of a sociometric type (Avramidis et al., 2017), might provide further insights into OCB-O behaviors.

### Limitations and future directions

A limitation was that participants self-reported their OCB rates in the present studies. Such reports are valid, and they may be more valid than other-reports of OCB (Carpenter et al., 2014), but it would still be valuable to determine whether individual differences in W-EI could be used to predict what coworkers or supervisors are capable of observing. Given the magnitude of the present associations, and given the links involving OCB-O rates, it is likely that coworkers or supervisors would perceive employees with higher W-EI levels to be more conscientious, dependable, and committed to their organizations.

In the future, too, it would be valuable to further probe mechanisms linking W-EI to OCB. Rioux and Penner (2001) have established that at least three different motives (prosocial values, organizational concern, and impression management) can guide citizenship behaviors and investigating potential links between these motives and the W-EI dimension would have merit. In addition, we have made the case that employees with higher W-EI levels are more engaged with the workplace and this suggestion would benefit from additional research that squarely focuses on engagement and its multiple manifestations (Rich et al., 2010). In this context, it may also be useful to examine perceptions of what counts as in-role behavior because employees with higher levels of W-EI may define their jobs in ways that encourage OCBs (Morrison, 1994).

### Conclusion

Being able to reason about emotions may be crucial in using them for productive purposes (Salovey and Mayer, 1990). The present studies have shown that abilities of this type, if they are contextualized for the workplace, are a robust predictor of tendencies toward organizational citizenship. These results reaffirm the value of emotional intelligence (in the form of W-EI) for successful workplace functioning.

## Data availability statement

The datasets presented in this study can be found in online repositories. The names of the repository/repositories and accession number(s) can be found at: https://osf.io/26tcu/?view_only=ec9f2275950c467ead7f4c16ee080451.

## Ethics statement

The studies involving human participants were reviewed and approved by NDSU Institutional Review Board. The patients/participants provided their written informed consent to participate in this study.

## Author contributions

MR, RI, and SK designed the studies and analyzed data. MR wrote the manuscript. RI and SK provided input. All authors contributed to the article and approved the submitted version.

## Conflict of interest

The authors declare that the research was conducted in the absence of any commercial or financial relationships that could be construed as a potential conflict of interest.

## Publisher’s note

All claims expressed in this article are solely those of the authors and do not necessarily represent those of their affiliated organizations, or those of the publisher, the editors and the reviewers. Any product that may be evaluated in this article, or claim that may be made by its manufacturer, is not guaranteed or endorsed by the publisher.
